# A missed diagnosis or a masquerading disease: back to the basics

**DOI:** 10.11604/pamj.2013.15.29.2039

**Published:** 2013-05-21

**Authors:** Rejani Lalitha, Christopher Kenneth Opio

**Affiliations:** 1 Department of Medicine, School of Medicine, College of Health Sciences, Makerere University, P. O. Box: 7072, Kampala, Uganda

**Keywords:** Pre-eclampsia, magnesium sulphate, pheochromocytoma, blood pressures

## Abstract

A 23-year-old gravid Ugandan female at 26 weeks was admitted to the maternity ward with sweats, abdominal pain, feeling of apprehension and palpitations. A diagnosis of pre-eclampsia was made and treatment with magnesium sulphate initiated. She was later transferred to intensive care unit for monitoring and control of blood pressure. Due to her labile blood pressures despite intravenous hydralazine and metoprolol, the pregnancy was terminated. However, she continued to have labile blood pressures. Better control of blood pressure was achieved on oral prazocin and nifedipine. The patient was then transferred to floor and discharged home a few days later. An abdominal computed-tomography scan showed a solid lobulated right paravertebral mass superio-medial to the right kidney. An open adrenelectomy was performed and antihypertensives discontinued. Histopathology revealed a benign pheochromocytoma. The mother had good post-operative outcome; however the premature baby died 2 days later in the special care unit.

## Introduction

Pheochromocytoma is rare during pregnancy however a strong index of suspicion is key to the diagnosis. Any hypertension presenting before 20 weeks of gestation should be thoroughly investigated. This case represents the lack of perception by many clinicians of other causes of hypertension in pregnancy. This patient had hypertension secondary to an adrenal tumor which was missed at initial presentation.

## Patient and observation

Patient and observation We present a 23-year old African female, a primigravida and an allied health professional. She did not have any major health issues other than an appendectomy 6 months before diagnosis of her current pregnancy at 8 Weeks of Gestation (WOG). Her first antenatal contact occurred at 12 WOG, during which she was diagnosed with hypertension in pregnancy. She complained of intermittent palpitations and headache. No intervention with anti-hypertensive drugs was required at that time. Additional details regarding this visit could not be obtained.

She continued to feeling unwell over the subsequent 3 months. Finally, at 26 WOG, she sought obstetric care at a large urban hospital. Her complaints at the time included intermittent progressive palpitations, feelings of apprehension, sweating, dizziness, tremulousness, headache and occasional abdominal pain. This was not associated with any urinary symptoms, fever, or any other related symptoms. She was not on any medication or recreational drugs. She did not smoke or consume alcohol during this pregnancy. Her Physical examination was remarkable for anxious looking facies, tachycardia of 105 to 130 beats per minute, and a high systolic Blood Pressure (BP) of 140 to 200 mmHg. Her admission BP was 200/120mmHg on both arms.The rest of her cardiorespiratory examination was normal. Fundoscopy was not done at admission. Her neurological examination was normal. Abdominal examination revealed a gravid uterus with fundal height at 24/40 weeks, no renal bruit was appreciated. Her pre-pregnancy Body Mass Index (BMI) was 23.5.

Investigations during this admission showed a normal complete blood counts and differentials, normal electrolytes including calcium, magnesium and phosphate (serum potassium of 3.9mmol/L), liver and renal panels. Urine dip stick was negative for proteinuria. Her random plasma glucose was 6.5mmol/L. An obstetric ultrasound done at admission showed a gravid uterus but no mention on the state of the kidneys, adrenal glands or presence of any abnormal abdominal masses. She was managed by her obstetrician as hypertension in pregnancy and started on intra muscular magnesium sulphate for her raised BP. However her hypertension was refractory to this treatment leading to the development of hypertensive urgency. She was transferred to the medical/surgical Intensive Care Unit (ICU) for monitoring and control of her BP. Further evaluation was not very helpful. Urinalysis and renal studies remained normal while Electrocardiogram (EKG) and echocardiogram showed features of left ventricular strain and moderate left ventricular hypertrophy. Fundoscopy showed bilateral Grade IV hypertensive retinopathy (papilledema). In ICU, her BP was managed with intravenous hydralazine and metoprolol. EKG monitoring was performed every 2 days for signs of myocardial ischemia in combination with daily assessment of symptoms and clinical signs for the same. Cardiac enzymes could not be done.

A decision to terminate the pregnancy at 27 WOG was made by obstetric team due to her labile BP with the hope that this would lead to better BP control and a live healthy baby. The cesarean section was uneventful leading to the birth of a premature neonate. Following the surgery, she was readmitted to ICU for monitoring. The baby died after 2 days in special care unit. She continued to experience swinging BP. This was controlled after titration with oral nifedipine and metoprolol. At this time, a diagnosis of secondary hypertension was strongly considered. Screening was undertaken for renal disease (imaging, renal function) and pheochromocytoma. A 24-hour urine metanephrine screen was negative but was inconclusive. Despite this a decision was taken to start her on medication for pheochromocytoma. The patient was transferred back to maternity with a diagnosis of secondary hypertension possibly due to pheochromocytoma and eventually discharged via the ward by the obstetric team/internists on oral prazocin 1mg once daily and oral nifedipine extended release 30mg twice daily. The plan was to continue her work up as an outpatient with further imaging and a repeat urinary vanillylmandelic acid (VMA).

**Figure 1:**
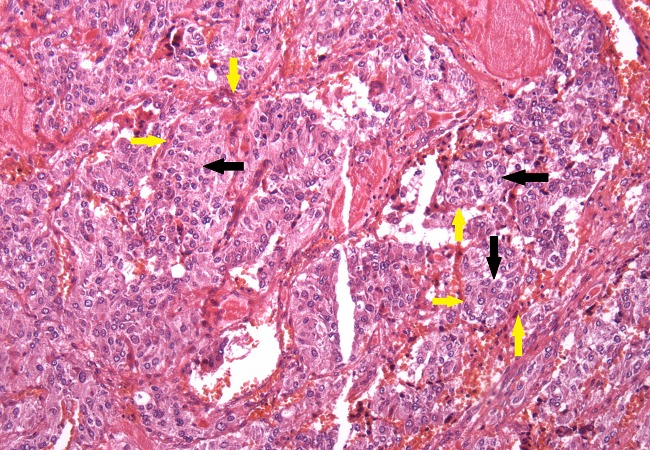
Section of tissue block obtained from adrenal tumor with Hematoxylin and Eosin stain, under low power, showing organoid neoplasm with large nests of tumor cells (black arrows) surrounded by rim of spindle cells or sustentacular cells (yellow arrows). These are features suggestive of an adrenal pheochromocytoma

**Figure 2:**
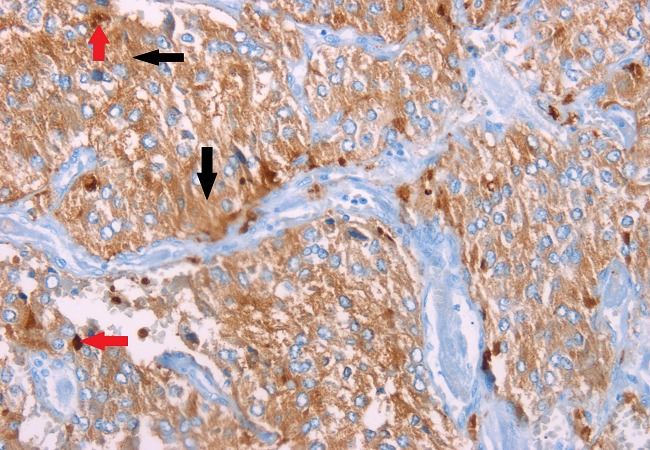
Immunohistochemistry of adrenal tumor showing diffuse cytoplasmic positivity of tumor cells to neuroendocrine markers namely Synaptophysin (black arrows) and Chromogranin (red arrows)

A chest Computed Tomography (CT) scan was performed and was normal. Abdominal CT scan showed a solid lobulated right paravertebral mass superior-medial to the right kidney with calcifications, extending from 11^th^, 12^th^ thoracic vertebrae to 13^th^ lumbar vertebra measuring 7.2 × 3.9 × 8.5 cm and compressing the inferior vena cava. The right supra renal gland was not visualized. The left supra renal gland and kidney were normal. A repeat 24 hour urinary VMA level was markedly elevated at 19.56 mg (normal range 2-7 mg/24h) with a normal creatinine clearance of 91.27 ml/min (normal range 88-128 ml/min) and 24h urine output of 2,450mls. This was sufficient to clinically confirm the diagnosis of pheochromocytoma.Her systolic BP was in the range of 150-160mmHg with oral prazocin 1mg twice daily and oral nifedipine 30mg twice daily. Oral propranolol 40mg twice daily was added to her treatment and liberal salt diet was encouraged in preparation for her surgery. She was readmitted after two weeks following her discharge for an open laparotomy and resection of the tumor. Before the surgery, her blood pressure was well controlled on the three antihypertensive drugs. The tumor was resected successfully and tissue samples were sent for histopathology using hematoxylin and eosin stain (Figure 1) and immunohistochemical staining (Figure 2). Following the surgery she spent two days in ICU for monitoring of BP and analgesia. At this point all antihypertensive were withdrawn and BP remained normal throughout her recovery and one year following her discharge from hospital.

### Differential diagnoses

Hypertension in pregnancy is a common problem thought to present in more than 8% of pregnancies [1]. It is classified into four categories namely: chronic hypertension, pre-eclampsia/enclampsia (hypertension and proteinuria after 20 WOG), pre-eclampsia superimposed on chronic hypertension and gestational hypertension (transient hypertension of pregnancy or chronic hypertension identified in the latter half of pregnancy). The patient described above falls into chronic hypertension, defined as hypertension (BP greater than 140/90mmHg) diagnosed at less than 20 WOG. Since the patient did not demonstrate any coexistent proteinuria/hyperuricaemia, it is unlikely that she had pre-eclampsia superimposed on chronic hypertension. The diagnosis of gestational hypertension is excluded since her hypertension was neither transient nor did it first present after 20 weeks of gestation.

One large study describing hypertensive disorders in pregnancy showed that chronic hypertension was twice as frequent as pre-eclampsia/eclampsia and 4 times more frequent than gestational hypertension or preeclampsia superimposed on chronic hypertension. In this study, chronic hypertension was twice more frequent in women of African ancestry compared to those with European or Asian origin [2]. In Uganda, one third of those with pre-eclampsia/eclampsia were shown to develop persistent hypertension; and risks factors for persistent hypertension after delivery included elevated serum creatinine, serum uric acid, and age group (20-29 years) [1]. One can postulate that many of these patients already had chronic hypertension.This collaborated by studies show the prevalence of chronic hypertension among females in this group in the community to be around 22% [3]. Chronic hypertension is mainly essential in nature, although it may be secondary to kidney diseases (primary or secondary), renal vascular diseases, adrenal diseases/disorders (primary aldosteronism, pheochromocytoma,Cushing's disease) and coarctation of the aorta. In reference to our patient, renal causes of hypertension were excluded at the time of admission on the basis of a normal creatinine, bland urinalysis and absence of renal bruit. Coarctation of aorta is unlikely based on the fact that she had the same blood pressure values on both the upper limbs and a normal cardiovascular examination. Cushing's syndrome or primary aldosteronism is also unlikely since the patient did not present with features diagnostic of Cushing's syndrome or laboratory findings of aldosteronism (low urine specific gravity, high urine PH, persistently low serum potassium and high urine potassium) except hypertension. In our patient a diagnosis of pheochromocytoma was confirmed on the basis of symptomatic paroxysmal persistent hypertension that is refractory to the usual drugs, adrenal mass, VMA greater than three times upper limit of normal, and complete remission of hypertension and removal of the need for antihypertensive medications following removal of tumor.

## Discussion

Pheochromocytoma is a rare catecholamine-secreting tumor and very rare in association with pregnancy. In 85% of patients, it is derived from chromaffin cells of adrenal medulla; the rest 15% arise from extra-adrenal locations. It is a rare cause of sustained and paroxysmal hypertension. Hypertension may alternate with hypotension if the tumor predominantly secretes epinephrine. The clinical manifestations of the disease such as palpitations, generalized inappropriate sweating, feelings of impending doom or apprehension and headache arise from catecholamines released by the tumor into the circulation. Several mechanisms have been described as to why the manifestations of pheochromocytoma become clinically overt only during and probably due to the pregnancy. The increased intra-abdominal pressure owing to expanding uterus, fetal movements hemorrhage into tumor, abdominal palpation e.g., during physical examination, uterine contractions, the process of delivery, and general anesthesia induce a surge of catecholamines. Peripartum period is especially precarious due to abrupt massive outpouring of catecholamines from stress of labor, anesthesia and vaginal delivery. Pheochromocytoma in pregnancy demands special attention as there are several unusual presentations of the disease which makes diagnosis difficult with grave consequences.

A high index of suspicion and a succinct history taking may help highlight the key symptoms and signs of pheochromocytoma, especially in our setting where resources are scarce, which warrants prompt evaluation. Reliable diagnosis requires a combination of biochemical tests and anatomical localization of the tumor by imaging studies. Plasma and 24-hour urinary metanephrine and normetanephrine are the most sensitive biochemical tests for making the diagnosis. Very rarely is clonidine suppression test indicated. Once the diagnosis is established, pheochromocytoma must be localized by appropriate imaging studies. A Computed Tomography (CT) can identify adrenal tumors which are 1cm or larger in 95% of the cases and localize 90% of extra-adrenal tumors larger than 2cm. Magnetic Resonance Imaging (MRI) is more sensitive and specific than CT. MRI is preferred in pregnant women and children suspected of having pheochromocytoma as it poses no radiation risk. MRI is currently not accessible by majority of the population in a low income country like ours. Tumor uptake of a radiopharmaceutical agent ^131^I- metaiodobenzylguanidine (MIBG) occurs in 81 to 85% of pheochromocytomas and is 95 to 100% specific for diagnosis. Imaging studies may be done before biochemical studies if familial pheochromocytoma is suspected where the tumor may not secrete significant amounts of catecholamines.

Treatment of pheochromocytoma involves timely management of hypertensive emergencies and urgency. Hydralazine, a direct arteriolar vasodilator, is used primarily to treat hypertensive crisis of pregnancy. One of the adverse effects is myocardial ischemia due to a reflex increases in heart rate and stroke volume especially in patients with pheochromocytoma but this effect can be reduced by combining the agent with a beta blocker like metoprolol after achieving appropriate alpha blockade. We used intravenous hydralazine and metoprolol as these agents are readily available in a resource limited setting unlike phentolamine or phenoxybenzamine. Phentolamine, an alpha blocker, is the drug of choice in treatment of hypertensive emergency in pheochromocytoma. Preoperative alpha adrenergic blockade with phenoxybenzamine or prazocin or calcium channel blockers can usually control pheochromocytoma hypertension. Preoperative β1 blockade may be needed to control supraventricular arrhythmias or tachycardias and angina.

Cardioselective β1 blocking agent like the long acting metoprolol is recommended for controlling supraventricular tachycardia. Alpha blockade should always be achieved before blocking the β1 receptors as beta blockade alone can cause marked hypertension. This is more common with non- selective beta blockers as these agents block β2 receptors inhibiting any vasodilatory effects of epinephrine and thereby promoting vasoconstriction. In our patient, preoperative alpha blockade was achieved by oral prazocin and nifedipine and beta blockade by propranolol as oral metoprolol is not available in Uganda although it is recommended to use cardioselective β1 blockers like metoprolol. Preoperative sedation with diazepam helps alleviate symptoms of anxiety and catecholamine release. A muscle relaxant is recommended before endotracheal intubation to prevent any surge of catecholamines. For intraoperative control of hypertension intravenous phentolamine, nitroprusside or nitroglycerine can be used. Arrhythmias can be controlled using intravenous esmolol and/or lidocaine. Proper intra-operative intravenous fluid administration minimizes any risk for post-operative hypotension. Resection of the tumor can be done laparoscopically or by open laparotomy. Open laparotomy is preferred when the tumors are multiple, very large or difficult to be removed laparoscopically. Preservation of the adrenocortical function is important to prevent lifelong steroid replacement therapy and its complications. Pheochromocytomas in pregnancy should be removed promptly but if the pregnancy is carried to term, delivery of the fetus should be done by caesarean section to avoid the stress of labor and vaginal delivery. An experienced surgeon and anesthesiologist are paramount to a successful resection of the tumor and favorable maternal-fetal outcomes.

## Conclusion

This case highlights the significance of a high index of suspicion and careful history taking in identifying these patients. This patient had hypertension secondary to an adrenal tumor which was missed at initial presentation. The importance of a broad differential diagnosis and special attention to diagnostic clues holds the key to right diagnosis and successful case management. We strongly recommend that clinicians should fully evaluate to determine other causes of hypertension among women presenting with raised blood pressure earlier than 20 weeks of gestation.
